# Oral anticoagulant decreases stroke recurrence in patients with atrial fibrillation detected after stroke

**DOI:** 10.3389/fcvm.2022.929304

**Published:** 2022-07-22

**Authors:** Jin-Yi Hsu, Peter Pin-Sung Liu, Luciano A. Sposato, Huei-Kai Huang, An-Bang Liu, Edward Chia-Cheng Lai, Swu-Jane Lin, Cheng-Yang Hsieh, Ching-Hui Loh

**Affiliations:** ^1^Center for Aging and Health, Hualien Tzu Chi Hospital, Buddhist Tzu Chi Medical Foundation, Hualien, Taiwan; ^2^School of Medicine, Tzu Chi University, Hualien, Taiwan; ^3^Institute of Medical Sciences, Tzu Chi University, Hualien, Taiwan; ^4^Department of Clinical Neurological Sciences, Schulich School of Medicine and Dentistry, Western University, London, ON, Canada; ^5^Heart and Brain Laboratory, Western University, London, ON, Canada; ^6^Department of Epidemiology and Biostatistics, Schulich School of Medicine and Dentistry, Western University, London, ON, Canada; ^7^Department of Anatomy and Cell Biology, Schulich School of Medicine and Dentistry, Western University, London, ON, Canada; ^8^Department of Family Medicine, Hualien Tzu Chi Hospital, Buddhist Tzu Chi Medical Foundation, Hualien, Taiwan; ^9^Department of Medical Research, Hualien Tzu Chi Hospital, Buddhist Tzu Chi Medical Foundation, Hualien, Taiwan; ^10^Department of Neurology, Hualien Tzu Chi Hospital, Buddhist Tzu Chi Medical Foundation, Hualien, Taiwan; ^11^School of Pharmacy, Institute of Clinical Pharmacy and Pharmaceutical Sciences, College of Medicine, National Cheng Kung University, Tainan, Taiwan; ^12^Department of Pharmacy Systems, Outcomes and Policy, College of Pharmacy, University of Illinois at Chicago, Chicago, IL, United States; ^13^Department of Neurology, Tainan Sin Lau Hospital, Tainan, Taiwan

**Keywords:** atrial fibrillation, atrial fibrillation detected after stroke, anticoagulant, ischemic stroke, intracranial hemorrhage

## Abstract

**Background:**

Atrial fibrillation detected after stroke (AFDAS) has a lower risk of ischemic stroke recurrence than known atrial fibrillation (KAF). While the benefit of oral anticoagulants (OAC) for preventing ischemic stroke recurrence in KAF is well established, their role in patients with AFDAS is more controversial. This study aimed to evaluate the association between OAC use and the risk of recurrent ischemic stroke in patients with AFDAS in a real-world setting.

**Methods:**

This nationwide retrospective cohort study was conducted using the Taiwan National Health Insurance Research Database. Patients hospitalized with a first-ever ischemic stroke and AFDAS confirmed within 30 days after hospitalization were assigned to OAC and non-OAC cohorts. Inverse probability of treatment weighting was applied to balance the baseline characteristics of the cohorts. The primary outcome was ischemic stroke recurrence. Secondary outcomes were intracranial hemorrhage (ICH), death, and the composite outcome of “ischemic stroke recurrence, ICH, or death.” Multivariate Cox proportional hazard models were used to estimate adjusted hazard ratios (aHR) and 95% confidence intervals (CI).

**Results:**

A total of 4,508 hospitalized patients with stroke and AFDAS were identified. Based on OAC use, 2,856 and 1,652 patients were assigned to the OAC and non-OAC groups, respectively. During the follow-up period (median duration, 2.76 years), the OAC cohort exhibited a lower risk of ischemic stroke recurrence (aHR, 0.84; 95% CI, 0.70–0.99), death (aHR, 0.65; 95% CI, 0.58–0.73), and composite outcome (aHR, 0.70; 95% CI, 0.63–0.78) than did the non-OAC cohort. The risk of ICH (aHR, 0.96; 95% CI, 0.62–1.50) was not significantly different between the two cohorts.

**Conclusion:**

OAC use in patients with AFDAS was associated with reduced risk of ischemic stroke recurrence, without an increased risk of ICH. This supports current guidelines recommending OACs for secondary stroke prevention in patients with AF, regardless of the time of diagnosis.

## Introduction

Stroke can be the initial clinical manifestation of previously undetected atrial fibrillation (AF) (1). Up to 58.7% of patients with AF-related acute ischemic stroke have AF detected after stroke (AFDAS) (2, 3). The prognosis and management of stroke patients with AFDAS have recently attracted more attention (3–9) owing to the increased utilization of advanced monitoring technology for AF screening after a stroke (10, 11). According to current guidelines (9, 12), newly detected AF in patients who suffered a stroke should prompt anticoagulation unless contraindicated. However, compared to patients with AF known before stroke (KAF), AFDAS seems to have a more benign profile (5, 6, 8, 13). A recent systematic review and meta-analysis showed that patients with AFDAS have a lower burden of risk factors, a lower CHA_2_DS_2_-VASc score, a smaller left atrium, and 26% lower risk of stroke recurrence than patients with KAF (14). Furthermore, another systematic review and meta-analysis of randomized controlled trials has shown that although prolonged cardiac monitoring in patients with stroke results increased AF detection and use of oral anticoagulants (OACs), it is not associated with reduced risk of stroke recurrence (15). These recent studies suggest that given the relatively benign risk profile of AFDAS, the use of OACs in these patients may not be as beneficial as it is for patients with KAF. However, to our knowledge, no prior randomized controlled trials or observational studies have confirmed the benefits of OACs in patients with AFDAS (16). Therefore, we conducted this nationwide population-based cohort study to examine the association between OAC use and ischemic stroke recurrence, as well as with intracranial hemorrhage (ICH) and death, in stroke patients with AFDAS.

## Materials and methods

### Data sources

The present study was conducted using data from Taiwan’s National Health Insurance Research Database (NHIRD) between 2000 and 2018. The NHIRD is derived from the electronic claims data of Taiwan’s National Health Insurance program, which enrolls more than 99% of the Taiwanese population (approximately 23.6 million). The NHIRD is currently stored and managed by the Health and Welfare Data Science Center of Taiwan’s Ministry of Health and Welfare (17). It provides comprehensive healthcare information, including medication prescriptions, medical device usage, and emergency, inpatient, or outpatient visits. Information on individual beneficiaries can be linked and longitudinally followed using an encrypted identification number. The study protocol was approved by the Institutional Review Board of Hualien Tzu Chi Hospital (IRB-107-152C). The requirement for obtaining informed consent was waived, as personal identifiers of patients were encrypted in the NHIRD.

### Study design, population, and definitions

**FIGURE 1:**
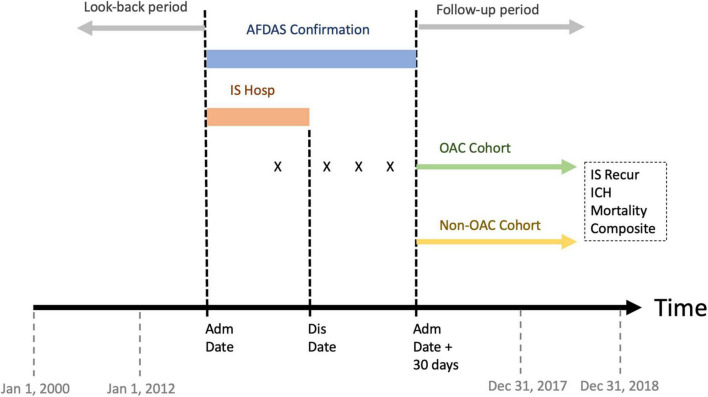
Study design. AFDAS, atrial fibrillation detected after stroke; Hosp, hospitalization; X, prescription of oral anticoagulant; Adm, admission; Dis, discharge; OAC, oral anticoagulant; IS Recur, ischemic stroke recurrence; ICH, intracranial hemorrhage.

In this retrospective cohort study, we identified consecutive adult patients hospitalized due to first-ever ischemic stroke with AFDAS between 2012 and 2017 (Figure 1). Each patient’s index date and year were defined as the admission date and year of the index stroke event, respectively. Ischemic stroke was defined based on ICD-9-CM codes 433 and 434 before 2016, and ICD-10-CM code I63 thereafter (18–20). ICH was defined by applying ICD-9-CM codes 430, 431, and 432 before 2016 and ICD-10-CM codes I60, I61, and I62 thereafter (21). Only patients with available brain imaging during hospitalization for their index stroke event were included.

We established a 10-year lookback window to identify and exclude patients with a previous diagnosis of stroke or related cerebral vascular disease (ICD-9-CM codes 430–438 or ICD-10-CM codes I60–I69), in either inpatient and outpatient claims, to avoid reporting bias based on outcomes and indication bias based on anticoagulant use. AF was identified by using ICD-9-CM codes 427.31 and ICD-10-CM code I48.0–I48.2 or I48.9 (22, 23). AFDAS was defined as a new diagnosis of AF in either the inpatient or outpatient claims within 30 days after the index date. For this purpose, we applied the same 10-year look-back window before the index date to exclude patients with a previous diagnosis of AF. In Taiwan, prolonged cardiac monitoring is not reimbursed by the National Health Insurance, so the vast majority of the AFDAS diagnoses are made on admission electrocardiography (ECG) or 24-h Holter. The diagnostic codes for ischemic stroke (18–20) and AF (22, 23) have been previously validated In Taiwan’s NHIRD.

We excluded patients with a previous diagnosis of severe valvular heart disease such as rheumatic heart disease (ICD-9-CM codes 393–398 or ICD-10-CM codes I00-I09), congenital heart disease (ICD-9-CM codes 746–747 or ICD-10-CM codes Q20-Q28), or those who had undergone valvular replacement surgery (NHI procedure code: 68016B, 68017B, 68018B). We also excluded patients who died or had new ischemic stroke or ICH within 30 days after the index date, prolonged hospitalization beyond 30 days, or age younger than 20 years (Supplementary Figure 1).

### Allocation of cohorts

The OAC cohort consisted of patients with first-ever ischemic stroke with AFDAS who received OACs within 30 days following the index date. The non-OAC cohort consisted of patients with AFDAS who never received OACs during the same 30-day period (Figure 1).

### Covariates

The baseline characteristics of both cohorts were listed in Table 1. The monthly income was defined based on the insurance premium, which was income-dependent and recorded on a graduated scale. It was categorized as dependent, USD 567–1,076, USD 1,077–1,615, and > USD 1,615. Comorbidities were defined as diagnostic codes recorded in at least one inpatient diagnosis or at least two outpatient diagnoses within 1 year before the index stroke event (23). These variables were also used to calculate the pre-stroke CHA_2_DS_2_-VASc scores (24). The timing of AFDAS was categorized as during the inpatient (before discharge) or the outpatient period (after discharge). Stroke severity was determined using a claims-based stroke severity index, which was further transformed to the estimated National Institutes of Health Stroke Scale (eNIHSS) score (25). We categorized the eNIHSS as mild (≤ 5), moderate (≥ 6 and ≤ 13), and severe (> 13) (26, 27). Other important covariates regarding the index stroke included length of hospitalization, physician specialty (neurology or others), and hospital level (tertiary referral center or others). To investigate anticoagulant use in the OAC cohort, we further classified patients into those treated with non-vitamin K antagonist oral anticoagulants for ≥ 1 day within the 30 days following the index date, and the others were defined as being treated with warfarin. Antiplatelet use was defined as the use of antiplatelet therapy for ≥ 1 day within the 30 days following the index date. 24-h Holter monitoring was defined as whether the patients received 24-h Holter monitoring within the 30 days following the index date.

**TABLE 1:** Baseline characteristics before and after IPTW.

	Original cohorts			IPTW cohorts		
	OAC	Non-OAC	SMD	OAC	Non-OAC	SMD
	*N* = 2,856	*N* = 1,652		*N* = 2,496	*N* = 1,434	
**Age**						
Age, years *	71.7 (11.7)	75.2 (11.9)	0.298	72.5 (10.8)	73.9 (11.2)	0.123
< 65	762 (26.7)	330 (20.0)	0.159	598 (24.0)	325 (22.7)	0.030
65–75	823 (28.8)	386 (23.4)	0.124	739 (29.6)	371 (25.9)	0.083
≥ 75	1,271 (44.5)	936 (56.7)	0.245	1,160 (46.5)	738 (51.4)	0.100
**Sex**						
Male	1,680 (58.8)	863 (52.2)	0.133	1,433 (57.4)	790 (55.1)	0.047
Female	1,176 (41.2)	789 (47.8)	0.133	1,063 (42.6)	644 (44.9)	0.047
**Index year** ^†^						
2012	389 (13.6)	331 (20.0)	0.172	363 (14.6)	245 (17.1)	0.070
2013	396 (13.9)	318 (19.3)	0.145	379 (15.2)	237 (16.5)	0.036
2014	454 (15.9)	308 (18.6)	0.073	427 (17.1)	264 (18.4)	0.035
2015	524 (18.4)	273 (16.5)	0.048	469 (18.8)	271 (18.9)	0.002
2016	541 (18.9)	208 (12.6)	0.175	424 (17.0)	204 (14.2)	0.076
2017	552 (19.3)	214 (13.0)	0.174	433 (17.4)	213 (14.8)	0.069
**Monthly income (USD)** ^‡^						
Dependent	762 (26.7)	468 (28.3)	0.037	676 (27.1)	403 (28.1)	0.023
567–1,076	1,364 (47.8)	853 (51.6)	0.077	1,229 (49.2)	728 (50.7)	0.030
1,077–1,615	373 (13.1)	187 (11.3)	0.053	323 (12.9)	170 (11.9)	0.032
> 1,615	357 (12.5)	144 (8.7)	0.123	268 (10.8)	133 (9.3)	0.049
**Comorbidities**						
Hypertension	1,513 (53.0)	919 (55.6)	0.053	1,334 (53.4)	775 (54.1)	0.013
Diabetes mellitus	580 (20.3)	349 (21.1)	0.020	514 (20.6)	300 (20.9)	0.008
Dyslipidemia	576 (20.2)	296 (17.9)	0.057	497 (19.9)	264 (18.4)	0.038
CAD	476 (16.7)	282 (17.1)	0.011	406 (16.3)	236 (16.4)	0.004
CHF	79 (2.8)	25 (1.5)	0.087	51 (2.0)	22 (1.6)	0.037
MI	37 (1.3)	38 (2.3)	0.075	28 (1.1)	24 (1.6)	0.044
**Pre-stroke CHA_2_DS_2_-VASc score** ^§^						
Score*	2.4 (1.4)	2.7 (1.4)	0.217	2.5 (1.4)	2.6 (1.4)	0.082
Low risk^‡^	394 (13.8)	168 (10.2)	0.112	300 (12.0)	166 (11.6)	0.013
Intermediate risk	560 (19.6)	244 (14.8)	0.129	483 (19.3)	253 (17.6)	0.045
High risk	1,902 (66.6)	1,240 (75.1)	0.187	1,713 (68.6)	1,016 (70.8)	0.047
**Timing of AFDAS diagnosis**						
Inpatient	2,541 (89.0)	1,427 (86.4)	0.079	2,219 (88.9)	1,264 (88.2)	0.024
Outpatient	315 (11.0)	225 (13.6)	0.079	277 (11.1)	170 (11.8)	0.024
**Stroke severity** ^| |^						
eNIHSS*	9.0 (6.1)	10.9 (7.1)	0.289	9.1 (6.0)	9.9 (6.6)	0.128
Mild^§^	1,525 (53.4)	741 (44.9)	0.172	1,297 (52.0)	718 (50.1)	0.038
Moderate	666 (23.3)	319 (19.3)	0.098	606 (24.3)	290 (20.2)	0.098
Severe	665 (23.3)	592 (35.8)	0.278	593 (23.8)	427 (29.8)	0.136
**Length of hospitalization**						
Days*	11.6 (7.5)	12.3 (8.0)	0.091	11.6 (7.4)	12.0 (7.9)	0.060
**Physician specialty**						
Neurology	2,517 (88.1)	1,348 (81.6)	0.183	2,208 (88.5)	1,228 (85.6)	0.086
Others	339 (11.9)	304 (18.4)	0.183	288 (11.5)	207 (14.4)	0.086
**Hospital level**						
Tertiary center	1,179 (41.3)	554 (33.5)	0.160	980 (39.3)	518 (36.1)	0.065
others	1,677 (58.7)	1,098 (66.5)	0.160	1,516 (60.7)	916 (63.9)	0.065
**Anticoagulant type**						
NOAC	1,855 (65.0)	n/a	n/a	1,585 (63.5)	n/a	n/a
Warfarin	1,001 (35.1)	n/a	n/a	912 (36.5)	n/a	n/a
**Antiplatelet use**						
Yes	1,687 (59.1)	1,055 (63.9)	0.099	1,492 (59.8)	939 (65.4)	0.117
No	1,169 (40.9)	597 (36.1)	0.099	1,004 (40.2)	496 (34.6)	0.117
**24-h Holter monitoring**						
Yes	1,226 (42.9)	614 (37.2)	0.118	1,055 (42.3)	565 (39.4)	0.059
No	1,630 (57.1)	1,038 (62.8)		1,441 (57.7)	869 (60.6)	0.059

Data are expressed as n (%) unless otherwise indicated.

*Expressed as mean (SD).

^†^Index year: the year of admission for the index stroke event.

^‡^1 NTD = 0.036 USD as of Nov 2021.

^§^CHA_2_DS_2_-VASc score: low stroke risk was defined as a score of 1 or 0 for women and 0 for men; intermediate stroke risk was defined as a score of 2 for women and 1 for men; high stroke risk was defined as a score of ≥ 3 for women and ≥ 2 for men.

^| |^Severity of stroke: mild severity was defined as a score of ≤ 5; moderate severity was defined as a score of ≥ 6 and ≤ 13; severe severity was defined as a score of > 13.

AFDAS, atrial fibrillation detected after stroke; CAD, coronary artery disease; CHF, congestive heart failure; eNIHSS, estimated National Institutes of Health Stroke Scale; IPTW, inverse probability of treatment weighting; MI, myocardial infarction; NOAC, non-vitamin K antagonist oral anticoagulant; OAC, oral anticoagulant; SMD, standardized mean difference.

### Follow-up and outcomes

The date of follow-up onset was defined as 30 days after the index date (Figure 1). This approach has been previously used (28) to avoid immortal time bias (29). That is, patients in both OAC and non-OAC cohorts have to survive up to the same starting time point to be included in the analysis of outcomes.

The primary outcome was ischemic stroke recurrence, defined as an inpatient diagnosis of ischemic stroke after an examination of brain imaging. The secondary outcomes included ICH, death, and a composite endpoint of “ischemic stroke recurrence, ICH, or death.” Death was defined by using the National Death Registry, linked to the Taiwan’s NHIRD (30).

### Statistical analysis

Categorical variables were expressed as counts and percentages, while continuous variables were expressed as means and standard deviations (*SD*). To minimize the selection bias inherent to a non-randomized controlled study, we used propensity score (PS) matching with a stabilized IPTW approach to create more homogeneous OAC and non-OAC groups with balanced baseline characteristics to facilitate comparisons. We calculated the PS using the logistic regression model and including covariates of age, sex, monthly premium level, pre-stroke CHA2DS2-VASc score, timing of AFDAS diagnosis, eNIHSS, length of hospitalization, physician specialty, hospital level, and comorbidities (listed in Table 1). The weights for the stabilized IPTW approach were defined as Z/PS for OAC group and (1-Z)/(1-PS) for the non-OAC group. Z and 1-Z were the marginal prevalence of OAC and non-OAC in the overall population, respectively. To avoid extreme weights, we removed patients whose PS were < 5% or > 95% of the population. Using PS with the stabilized IPTW approach could generate two interchangeable groups with the same treatment assignment probabilities, thus allowing for comparisons based on the average treatment effects of the entire population (31). Standardized mean differences were used to determine differences in baseline characteristics between the two cohorts, and a value of < 0.1 was considered no difference.

The probability of ischemic stroke event-free was estimated using the Kaplan-Meier method, and the difference between the event-free curves was examined using the log-rank test. The association between OAC use and primary and secondary outcomes was evaluated by applying multivariate Cox proportional hazard models and reported as hazard ratios (HR) and 95% confidence intervals (CI) (32). Multivariate models were adjusted for age, sex, income, comorbidities listed in Table 1, pre-stroke CHA_2_DS_2_-VASc score, timing of AFDAS diagnosis, eNIHSS, length of hospitalization, specialty of the treating physician (neurology or others), and hospital level (tertiary center or others).

Two sensitivity analyses were performed. First, a time-varying analysis was performed to account for crossovers in treatment groups during follow-up. Second, the Fine and Gray competing risk model was applied to account for the competing risk of ICH and death (33). Additionally, stratified analyses for age, sex, pre-stroke CHA_2_DS_2_-VASc score, timing of AFDAS diagnosis, eNIHSS, physician specialty, or hospital level were performed to estimate their interaction with the association between OAC use and the primary outcome. Statistical significance was defined as a two-tailed probability value of < 0.05. All statistical analyses were performed using SAS version 9.4 (SAS Institute Inc., Cary, NC) and Stata version 14.0 (StataCorp, College Station, TX).

## Results

### Baseline characteristics

A total of 4,508 hospitalized patients with both stroke and AFDAS were identified. Based on OAC use, 2,856 and 1,652 patients were assigned to the OAC and non-OAC groups, respectively. Patients in the OAC group tended to be younger, to have higher incomes and lower pre-stroke CHA_2_DS_2_-VASc and eNIHSS scores, and were more likely to be male, and to receive medical care from a neurologist or at a tertiary center (Table 1). In the IPTW cohorts, the baseline characteristics were well balanced between the two groups, except that the OAC group tended to be younger, had lower eNIHSS scores, and lower proportions of severe stroke, antiplatelet use than did the non-OAC group.

### Primary and secondary outcomes in IPTW cohorts

**FIGURE 2:**
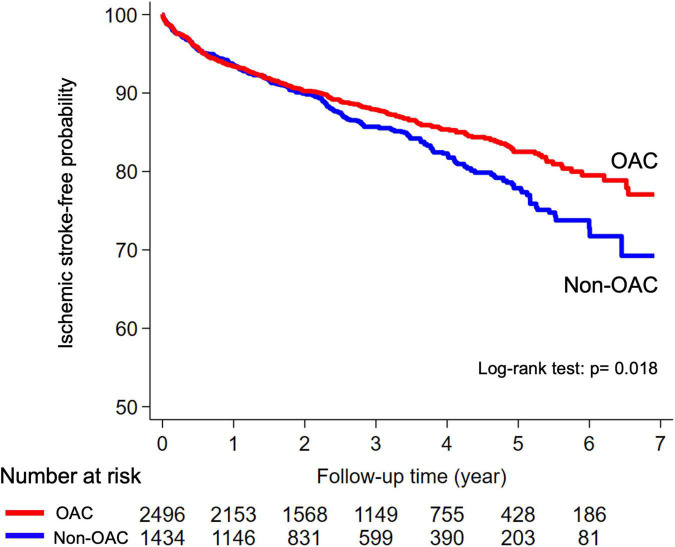
Kaplan–Meier curves for ischemic stroke event-free probability in the OAC and non-OAC cohorts among patients with AFDAS. AFDAS, atrial fibrillation detected after stroke; OAC, oral anticoagulant.

In the non-adjusted analysis, the risk of ischemic stroke recurrence was lower in the OAC cohort than in the non-OAC cohort (log-rank test, *p* = 0.018; Figure 2). At a median follow-up of 2.76 and 2.53 years, respectively (Table 2), the numbers (annualized event rates) of ischemic stroke recurrences in the OAC and non-OAC cohorts were 321 (4.29%) and 209 (5.33%), respectively. The univariate Cox proportion hazard model indicated a significantly lower risk of ischemic stroke recurrence in the OAC cohort than in the non-OAC cohort (HR, 0.81; 95% CI, 0.69–0.97; *p* = 0.018). This association remained significant in the multivariate model (adjusted HR, 0.84; 95% CI, 0.70–0.99; *p* = 0.042) (Table 2). Patients in the OAC cohort had a similar risk of ICH (adjusted HR, 0.96; 95% CI, 0.62–1.50; *p* = 0.864), and had a lower risk of death (adjusted HR, 0.65; 95% CI 0.58–0.73; *p* < 0.001) and the composite outcome (adjusted HR, 0.70; 95% CI, 0.63–0.78; *p* < 0.001), compared to patients in the non-OAC cohort.

**TABLE 2:** Risk of ischemic stroke and secondary outcomes in IPTW cohorts.

	Event	FU*	AER^†^	Univariate model			Multivariate model^‡^		
				HR	95% CI	*p*	aHR	95% CI	*p*
**Ischemic stroke**									
OAC	321	2.76	4.29	0.81	0.69–0.97	0.018	0.84	0.70–0.99	0.042
Non-OAC	209	2.53	5.33	Ref.					
**Intracranial hemorrhage**									
OAC	55	2.76	0.73	0.96	0.62–1.49	0.861	0.96	0.62–1.50	0.864
Non-OAC	30	2.53	0.76	Ref.					
**Death**									
OAC	600	3.11	7.29	0.57	0.51–0.64	<0.001	0.65	0.58–0.73	<0.001
Non-OAC	557	2.84	12.90	Ref.					
**Composite outcome^§^**									
OAC	825	2.76	11.02	0.64	0.58–0.71	<0.001	0.70	0.63–0.78	<0.001
Non-OAC	680	2.53	17.29	Ref.					

*Expressed as median duration of follow-up (years).

^†^Expressed as annualized event rate (%).

^‡^Hazard ratios were calculated using multivariate Cox regression models with adjustment for age, sex, index year, monthly income, comorbidities listed in Table 1, pre-stroke CHA_2_DS_2_-VASc score, diagnosis of AFDAS, eNIHSS score, length of hospitalization, physician specialty, and hospital level.

^§^Composite outcome defined as development of ischemic stroke, intracranial hemorrhage, or mortality.

aHR, adjusted hazard ratio; AER: annualized event rate; CI, confidence interval; eNIHSS, estimated National Institutes of Health Stroke Scale; FU, follow-up; HR, hazard ratio; IPTW, inverse probability of treatment weighting; IR, incidence rate; OAC, oral anticoagulant.

### Sensitivity analyses

In the time-varying sensitivity analysis accounting for treatment group crossovers, OAC use was associated with a nearly 50% lower risk of ischemic stroke recurrence (adjusted HR, 0.52; 95% CI, 0.43–0.63; *p* < 0.001) (Table 3). In Fine and Gray’s competing risk model, OAC use was also associated with a similar trend of lower risk of stroke recurrence compared with non-OAC use (adjusted HR, 0.91; 95% CI, 0.76–1.06; *p* = 0.305) (Table 3).

**TABLE 3:** Sensitivity analyses in the risk of ischemic stroke in IPTW cohorts.

	Univariate model			Multivariate model		
	HR	95% CI	*p*	aHR^†^	95% CI	*p*
Sensitivity analysis A*						
OAC	0.55	0.47–0.66	<0.001	0.52	0.43–0.63	<0.001
Non-OAC	Ref.			Ref.		
**Sensitivity analysis B^†^**						
OAC	0.90	0.76–1.07	0.240	0.91	0.76–1.09	0.3050
Non-OAC	Ref.			Ref.		

*Sensitivity analysis A: we used time-varying analysis to evaluate the effect of OAC on the primary outcome.

^†^Sensitivity analysis B: we used the Fine and Gray’s competing risk model to evaluate the effect of OAC on primary outcome.

aHR, adjusted hazard ratio; CI, confidence interval; HR, hazard ratio; IPTW, inverse probability of treatment weighting; OAC, oral anticoagulant.

### Stratified analysis

In stratified analysis, there was no significant interaction for age, sex, pre-stroke CHA_2_DS_2_-VASc score, timing of AFDAS diagnosis, 24-h Holter monitoring, eNIHSS, physician specialty, or hospital level with the association between OAC and stroke recurrence (Supplementary Table 1).

## Discussion

In this large population-based retrospective cohort study, the use of OACs in patients with first-ever ischemic stroke and AFDAS was associated with a 16% lower risk of ischemic stroke recurrence during a median follow-up of 2.76 years. Results were consistent in sensitivity analyses accounting for treatment group crossovers and the compering risk of ICH and death. There were no differences in the risk of ICH between treatment groups. There were no significant interactions identified for age, sex, CHA_2_DS_2_-VASc score, timing of AFDAS diagnosis, 24-h Holter monitoring, eNIHSS, physician specialty, or hospital level.

Currently, major guidelines suggest the use of OAC in patients with stroke and AF, without differentiating between KAF or AFDAS (9, 12). This is mainly based on the fact that AFDAS is a fairly novel concept (13, 15), and that there have not been any specific randomized clinical trials of OACs vs. antiplatelet agents or no antithrombotic therapy in patients with AFDAS. The results of the present real-world population-based study represent the closest possible approach to filling this knowledge gap, since a randomized controlled trial of OACs would be ethically unfeasible.

It is important to note that not all AFDAS have the same embolic risk. It has been proposed that AFDAS identified on the admission ECG or on short-term monitoring (e.g., 24-h Holter) may entail a higher burden and embolic risk, whereas lower-burden AFDAS detected on prolonged cardiac monitoring (e.g., 30-day external loop recorders or 2 or 3-year implantable loop recorders) may lower the risk of stroke recurrence (15). In the present study, AFDAS was diagnosed on admission with ECGs or 24-h Holter monitoring within 30 days after stroke in usual care settings. As a result, most AFDAS may have been high-burden and may have occurred asymptomatically before stroke occurrence. Although this assumption is hypothetical, the likely high-burden nature of most AFDAS in our cohorts may explain the association between OAC use and lower risk of stroke recurrence.

In sensitivity analysis, the time-varying analysis accounting for changes in OAC exposure during the follow-up period found that there was an even greater risk reduction (nearly 50% reduction in HR, *p* < 0.001) in ischemic stroke recurrence than there was in the main analysis (16% reduction, in HR, *p* = 0.042). However, this association was not statistically significant after taking into account the competing risks of ICH and death using Fine and Gray’s method in sensitivity analysis (9% reduction in HR, *p* = 0.305). This highlights the importance of adherence to OAC treatment for patients with AFDAS, and this information might provide physicians more confidence to initiate and maintain OAC treatment for post-stroke care in these patients. As only 37.1% and 39.3% of patients with stroke and newly confirmed AFDAS on serial ECGs or 24-h Holter monitoring, respectively, were prescribed with OACs at discharge (34), our real-world evidence lends support to current guidelines and indicates that physicians could prescribe OAC early with confidence once AFDAS has been confirmed.

### Limitations

Our study has several limitations. First, the diagnosis of AFDAS in the present study was mainly based on ECGs at admission and 24-h Holter monitoring. As such, the results are not generalizable to patients with AFDAS on prolonged Holter monitoring or implantable loop recording, who may have a different (and probably lower) AF burden. Results are awaited from those ongoing randomized trials, such as the FIND-AF2 trial (35), which is expected to provide more definitive information on this subject. Second, the use of a limited time window (30 days after the index stroke event) to identify the OAC and non-OAC cohorts is a limitation of the current study, because there could be cross-overs between the specified time windows. Third, unmeasured confounders such as hemorrhagic transformation, the size of cerebral infarctions, cerebral microbleeds, or comorbidities associated with high embolic or hemorrhagic risk may have influenced the results. However, the application of IPTW, as well as the consistency of the results of multivariate models and sensitivity analyses, suggest that our results are unlikely to be explained by selection bias. Fourth, the proportion of severe stroke (eNIHSS > 13) was higher in the non-OAC group, even after the application of IPTW. Nevertheless, the *p*-value for this interaction was insignificant (*p* = 0.224) for the severe stroke subgroup (Supplementary Table 1). Fifth, the use of a 10-year lookback period to exclude patients with a previous stroke and/or a previous AF diagnosis may have led to misclassification. However, this risk might be negligible (5, 36). Sixth, it would be more accurate to consider a certain proportion of patients who were re-admitted within the first 30-day period after index stroke admission as experiencing a continuation of the same stroke episode, instead of having an early stroke recurrence. Excluding these patients from the current study may have caused a selection bias. Lastly, we did not apply a cut-off value for AF duration for it to be considered as clinically relevant. AF was identified retrospectively based on claims records (22, 23). Such AF was likely to be high burden, because it was diagnosed on admission ECGs or short-term monitoring in usual care; therefore, it was probably a fairly homogenous group of AFDAS from a prognostic perspective.

## Conclusion

For acute patients with ischemic stroke with AFDAS, OAC initiation within 30 days after stroke was associated with a reduced risk of ischemic stroke recurrence but without a significantly increased risk of ICH. This finding might support current guidelines that recommend the use of OAC for secondary stroke prevention in patients with AF, regardless of AFDAS or KAF.

## Data availability statement

Taiwan’s NHIRD is maintained and regulated by the Health and Welfare Data Science Center at the Ministry of Health and Welfare in Taiwan. The dataset only could be utilized in the division of the Health and Welfare Data Science Center. Researchers who are interested to analyze this dataset can request access to the Taiwan Ministry of Health and Welfare. Requests to access the datasets should be directed to Taiwan Ministry of Health and Welfare (website: https://dep.mohw.gov.tw/DOS/cp-2516-3591-113.html).

## Ethics statement

The studies involving human participants were reviewed and approved by the Hualien Tzu Chi Hospital. Written informed consent for participation was not required for this study because personal identifiers of patients were encrypted in the NHIRD.

## Author contributions

J-YH: manuscript preparation, study conception and design, data extraction, and interpretation. PP-SL: study design and data extraction statistical analysis. LAS and S-JL: critical revision of the manuscript. H-KH: study conception and design and data interpretation. A-BL: study conception and data interpretation. EC-CL: statistical consultation and data interpretation. C-YH: study conception and design, data interpretation, and critical revision of the manuscript. C-HL: study conception and design and critical revision of the manuscript. All authors contributed to the article and approved the submitted version.

## Conflict of interest

The authors declare that the research was conducted in the absence of any commercial or financial relationships that could be construed as a potential conflict of interest.

## Publisher’s note

All claims expressed in this article are solely those of the authors and do not necessarily represent those of their affiliated organizations, or those of the publisher, the editors and the reviewers. Any product that may be evaluated in this article, or claim that may be made by its manufacturer, is not guaranteed or endorsed by the publisher.
