# Supplementary material for: Oral anticoagulant decreases stroke recurrence in patients with atrial fibrillation detected after stroke
Source: Front Cardiovasc Med. 2022 Jul 22;9:929304. doi: 10.3389/fcvm.2022.929304 (PMC9354040; doi:10.3389/fcvm.2022.929304)
Supplement: Supplementary file 1 [file Data_Sheet_1.pdf]

Supplementary Materials

Supplemental Figure S1. Study sample flow-chart. Abbreviation: AF, atrial fibrillation; AFDAS, atrial fibrillation detected after ischemic stroke; NHIRD, national health insurance research database; IPTW, inverse probability of treatment weighting; OAC, oral anticoagulants.

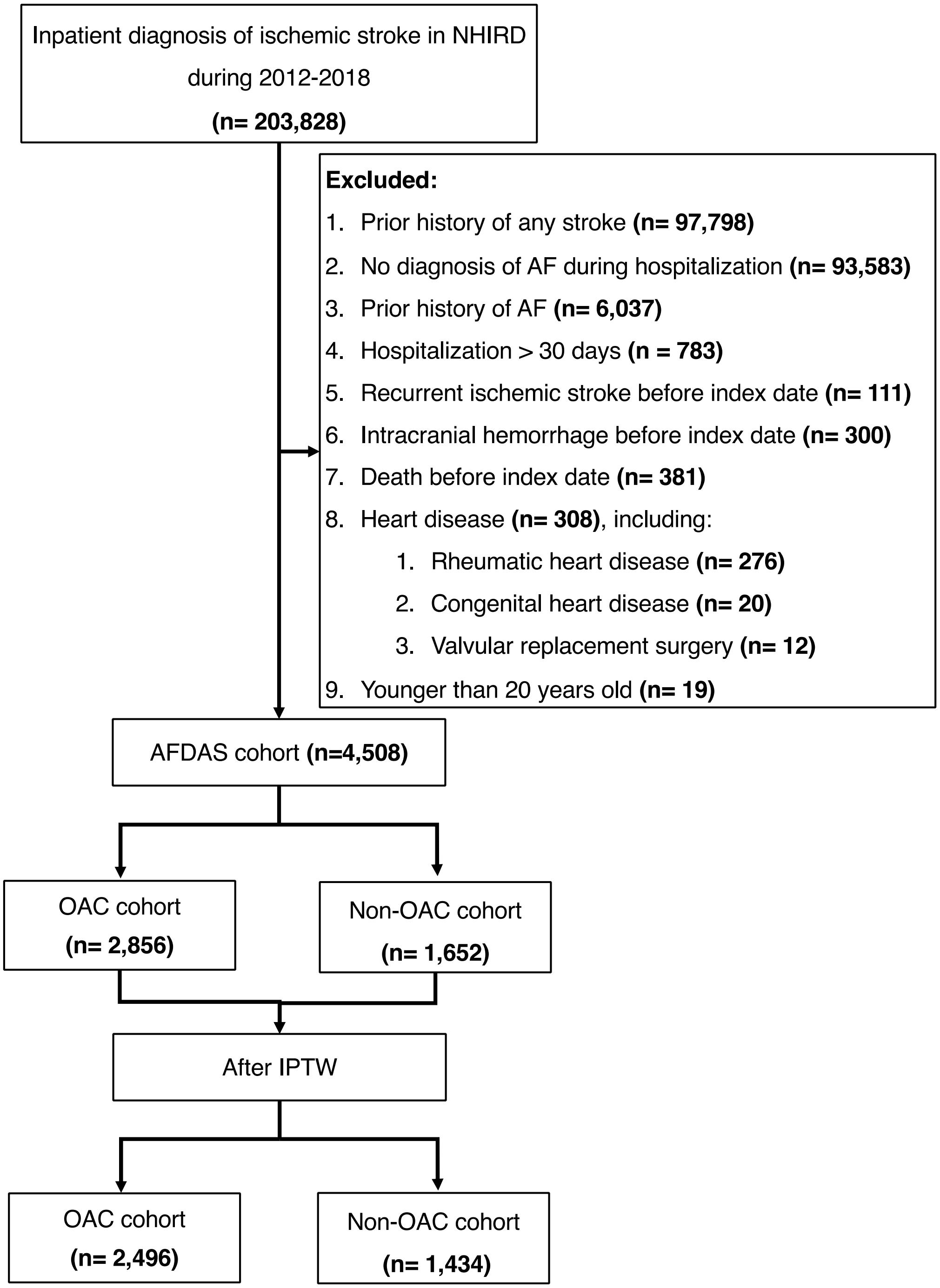

Supplemental Table S1. Stratified analysis for the assessment of the risk of ischemic stroke recurrence in IPTW cohorts

	aHR*	95% CI	*p*-value	*p* for interaction
**Age, years**				
<65	0.96	0.66–1.41	0.835	Reference
65–75	0.81	0.59–1.10	0.181	0.423
≥75	0.81	0.63–1.05	0.110	0.561
**Sex**				
Male	0.77	0.61–0.97	0.023	0.326
Female	0.95	0.73–1.25	0.735	
**Pre-stroke CHA_2DS_2-VASc score†**				
Low risk†	0.76	0.42–1.38	0.367	Reference
Intermediate risk	1.02	0.68–1.53	0.925	0.295
High risk	0.78	0.63–0.95	0.015	0.702
**Timing of AFDAS diagnosis**				
Inpatient	0.83	0.69–1.00	0.049	0.701
Outpatient	0.88	0.51–1.52	0.639	
**24-hour Holter monitoring**				
Yes	0.70	0.53-0.93	0.015	0.137
No	0.94	0.75-1.17	0.550	
**Severity of Stroke‡**				
Mild (eNIHSS ≤5)	0.82	0.65–1.03	0.083	Reference
Moderate (eNIHSS 6−13)	0.66	0.45–0.97	0.034	0.318
Severe (eNIHSS >13)	1.18	0.79–1.75	0.421	0.224

**Physician specialty**				
Neurology	0.80	0.67–0.97	0.019	0.340
Others	1.00	0.59–1.71	1.000	
**Hospital level**				
Tertiary center	0.84	0.62–1.15	0.276	0.884
Others	0.83	0.68–1.03	0.091	

* Hazard ratios were calculated using the non-OAC cohort as the reference group in multivariate Cox regression models with adjustment for all factors included in the main analysis.

† CHA_2DS_2-VASc score: high stroke risk was defined as a score of ≥3 for women and ≥2 for men; intermediate stroke risk was defined as a score of 2 for women and 1 for men; low stroke risk was defined as a score of 1 or 0 for women and 0 for men.

‡ Severity of stroke: mild severity was defined as a score of ≤ 5; moderate severity was defined as a score of ≥ 6 and ≤ 13; severe severity was defined as a score of > 13.

Abbreviation: AFDAS, atrial fibrillation detected after stroke; aHR, adjusted hazard ratio; CI, confidence interval; eNIHSS, estimated National Institutes of Health Stroke Scale; OAC, oral anticoagulant. IPTW, inverse probability of treatment weighting
